# Analysing the use of music to facilitate social interaction in care home residents with dementia: Narrative synthesis systematic review

**DOI:** 10.1177/14713012221100625

**Published:** 2022-05-20

**Authors:** Bryony Waters, Lídia Sousa, Martin Orrell, Orii McDermott

**Affiliations:** School of Medicine, Institute of Mental Health, 6123 University of Nottingham, UK; Faculty of Medicine of Porto University, 59043 Center of Health Technologies and Services Research - CINTESIS, Portugal; School of Medicine, Institute of Mental Health, University of Nottingham, UK; School of Medicine, Institute of Mental Health, 6123 University of Nottingham, UK

**Keywords:** dementia, social interaction, social behaviour, care homes, care staff, music therapy, music activities

## Abstract

**Introduction:**

Individuals with dementia residing in care homes can rely heavily on care staff to access activities and meaningful interactions. Previous research suggests that care home interactions can be short, fragmented and task-orientated due to staff workload and residents’ language impairments. However, music has the potential to be an alternative communication form that remains intact in the later stages of dementia. This systematic review aims to explore how care home music interventions can facilitate social interactions.

**Methods:**

A narrative synthesis was conducted to explore the mechanisms behind how and why care home music intervention facilitate social interactions. The four-element framework guided analysis; (1) Developing a theory, (2) Developing a preliminary synthesis, (3) Exploring relationships, (4) Assessing robustness.

**Findings:**

The final synthesis included 23 articles. The studies consisted of music therapy sessions, personalised music listening, structured music singing or instrument playing sessions and music therapeutic care. Despite the difference in music interventions, most studies reported an increase in residents’ sociable verbal and non-verbal communication and a decrease in unsociable communication. Music interventions allowed residents to reminisce, express themselves, focus and connect with others.

**Discussion:**

The studies highlighted music interventions are accessible to all residents with dementia despite their impairments. The adaptability allows individuals to continue to connect and express themselves even when language deteriorates. More research is needed into the enablers and barriers to implementing interventions into practice, as this systematic review has highlighted that some form of music intervention for all residents can be highly beneficial. Care homes use of music could increase social interactions and meaningful activities.

## Introduction

In the UK, approximately 250,000 individuals with dementia reside in care homes (Alzheimer’s Society, 2016), Comprising of 70% of all residents. Transitioning into a care home can be stressful and upsetting, as many feel they are losing their home, identity and independence (Thein et al., 2011). The transition can be especially frightening for individuals with dementia, whose impairments such as memory loss may make the care home seem scary, confining and isolated (Wiersma & Pedlar, 2008).

People in care homes can rely heavily on carers for assistance with daily tasks, accessing activities and meaningful interactions. Relationships developed between carers and residents impacts residents’ well-being and quality of care (Willemse et al., 2015). Residents with dementia can experience a lack of stimulating interactions or activities (Schreiner et al., 2005), and when interactions do occur, they have been characterised as short, fragmented and task-orientated, with little resident participation (Kolanowski & Litaker, 2006; Savundranayagam, 2014; Ward et al., 2008). Willemse et al. (2015) found that residents experienced 1.5 meaningful interactions over a 6-h observation period, with one-third of participants experiencing no positive interactions at all. The limited interactions could result from the demands on carers, who generally have excessive workloads and limited available time. Workload pressures can lead to residents' physical health and safety being the exclusive priority, with psychological, social and emotional needs being left unmet (Moyle et al., 2013). Not only can interactions be complicated due to staff workload, but many individuals with dementia can also experience a considerable reduction in verbal communication due to cognitive functioning impairments. As dementia progresses, individuals may have limited verbal abilities to express themselves. As a result, they may retreat from social interactions when impairment initially develop due to embarrassment and frustration (Banovic et al., 2018; Veselinova, 2014). Communication is not solely verbal; individuals can use non-verbal elements such as eye contact, smiling, touch, gestures and behaviours (Parker, 2003). However, these more subtle interactions can go unnoticed or are misinterpreted by staff (Ward et al., 2008).

Music can support communication, even when language deteriorates (Cross, 2014). Music processing is evident in early human development, with babies recognising musical elements (Zatorre, 2005), and it would seem humans continue to process music throughout their lives. The brain areas associated with music processing seems to remain intact throughout the stages of dementia when other areas of the brain that control cognitive functions such as memory and language deteriorate (Brotons & Koger, 2000; Jacobsen et al., 2015; Peretz & Coltheart, 2003). Unlike language, which is processed mainly in the left hemisphere, music processing requires nearly every brain area (Levitin, 2008; Peretz & Coltheart, 2003). Music can be enjoyed by individuals of all ages, ethnicities and many health conditions. The adaptability and accessibility of music makes it a potentially useful non-pharmacological intervention for people with dementia (Sacks, 2011). In dementia care, two types of music interventions are commonly used in care homes, music therapy and music activities. Music therapy is delivered by a registered Health and Care Professions Council (HCPC) music therapist (Schneider, 2018). It involves developing therapeutic relationships between the therapist and patient through listening and responding to sounds (McDermott et al., 2013). The content of musical activities varies more; they are generally facilitated by musicians, volunteers, family members, carers or activity coordinators and consist of various activities, including recorded music listening, singing, musical instrument playing and live music performances. Music therapeutic care is also encompassed under the umbrella of music activities; this generally involves music during care tasks (Götell et al., 2002). Both types of interventions can consist of singing, music listening, dancing, instrument playing and song writing. Previous research has highlighted a range of therapeutic benefits for individuals with dementia both in the community and care homes, including reduced agitation, anxiety, depression, increased alertness and maintaining an identity (Bannan & Montgomery-Smith, 2008; Hammar, 2011c; Hays & Minichiello, 2005; Ing-Randolph et al., 2015; Ray & Götell, 2018; Ridder et al., 2013). The benefits of music for individuals with dementia have been observed in both formal music therapy interventions and informal music activities. These benefits can aid residents to remain socially active, express themselves, and maintain a high quality of life and care whilst living in the care home.

Whilst many systematic reviews have explored the effects of music, many focus exclusively on music therapy, or not solely social interaction outcomes (Brotons et al., 1997; Fusar-Poli et al., 2018; McDermott et al., 2013; Petrovsky et al., 2015; Van der Steen et al., 2018; Vink & Hanser, 2018). To our knowledge, this is the first systematic review to explore how care home music interventions may facilitate social interactions, including both quantitative and qualitative studies. The data collected, identified previously researched interventions and their efficacy, which can aid the development of future music interventions to improve care home interactions. This review investigates the quality, efficacy and care home music intervention mechanisms to facilitate social interactions. This review will also explore whether the mechanisms for social interactions differ for music therapy compared to music activities

## Methods

This review was conducted using a prespecified protocol and in accordance with the Preferred Reporting Items for Systematic Reviews and Meta-analyses (PRISMA) statement (Moher et al., 2009). A narrative synthesis was conducted due to the flexibility of the approach, allowing for the exploration of the mechanisms for how and why care home music interventions facilitate social interactions, in studies with considerable heterogeneity in methodology, interventions and participants (Popay et al., 2006). Popay et al.’s four-element framework was used to answer the following research question: How do care home music interventions facilitate social interactions for residents with dementia.

Popay’s et al. (2006) four-element framework

(1) Developing a theory of how the intervention works, why and for whom (Presented in Methods)
(2) Developing a preliminary synthesis of findings of included studies (Presented in Findings)
(3) Exploring relationships in the data (Presented in Findings and Discussion)
(4) Assessing the robustness of the synthesis (Presented in Findings and Discussion)

### Narrative synthesis element one: Developing a theory

The aims of music therapy and music activities differ. Music therapy is driven by achieving therapeutic goals and developing therapeutic relationships to address an individual’s psychological, social and emotional needs (American Music Therapy Association, 2021; British Association for Music Therapy, 2017). Whereas music activities are generally used for health promotion or recreational goals (Stegemann et al., 2019). Although the aims differ, previous research highlights that benefits can be similar if implemented successfully.

**Figure 1.**
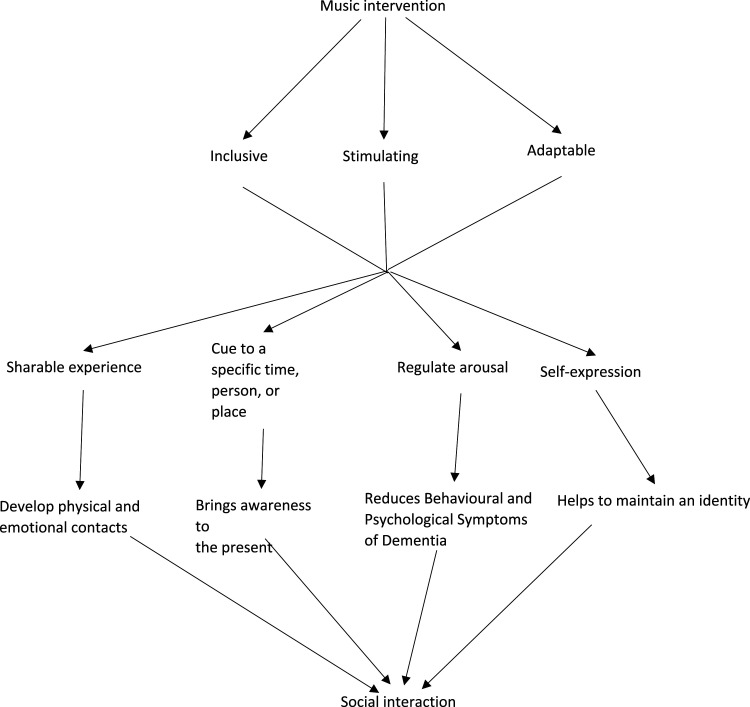
Model created based on element 1 theory development highlighting how music interventions may help to facilitate social interactions. The model presents the authors interpretation of the literature prior to the literature search.

McDermott’s et al. (2014) Psychosocial Model of Music in Dementia explored the importance of music to individuals with dementia. From the model, several potential mechanisms have been highlighted to explain why music may facilitate social interactions. The model consists of three overlapping concepts highlighting the importance of music for individuals with dementia: ‘who you are’, ‘here and now’ and ‘connectedness’. When cognitive functions decline, music can help individuals maintain an identity and connection to their life history. The stimulating nature can orientate the individual to the present and allow self-expression. The adaptability enables activities to be inclusive through altering the complexity and involvement. Music is a shareable experience that can aid physical and emotional contact. Additional potential mechanisms for music facilitating interactions include music as cues and to regulate arousal when individuals are in a state of agitation, confusion or disinterest (Allan, 2012; Ridder, 2003; Sixsmith & Gibson, 2007). McDermott’s et al. (2014) model is a broad view of the mechanisms of music for individuals with dementia; the current review will explore in-depth the mechanisms highlighted in McDermott’s model to gain insight into how music interventions in care homes may facilitate social interactions. Figure 1 presents a model developed by the authors to interpret the literature prior to the literature search. The model displays the author’s theory development highlighting how music interventions may help facilitate social interactions. Element 1 developing a theory has guided the research question, literature search and inclusion criteria.

For this review, music intervention will refer to any activity, active or passive, provided in care homes, where the main element is musical. The activities could be provided by care staff, researchers, musicians or music therapists. Social interactions will refer to exchanges between two or more individuals, including verbal and non-verbal communication and other social behaviours such as sitting together.

### Literature search

A literature search was conducted from the earliest date available to March 2020 using the electronic databases Embase, Medline, PubMed, Cinhahl, Cochrane library, Web of Science and PsycINFO on the 5^th^ March 2020. The search was re-run on the 22^nd^ July 2021. In addition, a hand search of included papers’ reference lists was conducted.

The following search terms were used:

• (Dement* OR Alzheimer* or Frontotemporal dementia OR Lewy body dementia)
• (Music OR Music Therap* OR Singing OR Sing OR Musical*)
• (Interaction* OR Social interaction OR Relationship* OR Social exchange OR Communication OR Non-verbal* OR Social behaviour OR Interpersonal interaction)

### Inclusion criteria

Articles were required to fulfil all inclusion criteria to be eligible.

• Adults aged 18+ with a diagnosis of dementia residing in care homes.
• Studies focussing on care staff were included if the intervention involved the participation of residents.
• The intervention’s main element was musical, facilitated by care staff, musicians, researchers or music therapists.
• Reported outcomes of social interactions, relationships or social behaviours
• Quantitative and/or Qualitative methods including- RCT, Case studies, Non-randomised, Observation, Interviews, and Before and After
• Written in English
• Published in a peer-reviewed journal

There was no restriction on gender, dementia type or severity.

### Exclusion criteria

• Participants with mild cognitive impairments
• Studies conducted in the community or consisted of both community-dwelling and resident participants
• Systematic reviews, Book chapters, Theses, Conference papers, Opinion pieces and Commentaries

### Article screening

After removing duplicates, articles were screened for eligibility in a two-stage process, title and abstract, then full text. Two reviewers (BW and LS) independently screened the articles and documented eligibility and exclusion reasons. Any discrepancies were discussed between reviewers, and unresolved discrepancies were resolved with a third reviewer (OM). Authors were contacted if papers were inaccessible.

### Methodology quality check

The methodology quality of included articles was assessed independently by the two reviewers using the Downs and Black (1998) and Critical Appraisal Skills Programme (CASP) (2018) tools. These tools were selected on the recommendations from Deeks et al. (2003) and the Centre for Reviews and Dissemination (2009).

Downs and Black (1998) assessed randomised and non-randomised quantitative studies, consisting of 27 questions across five domains reporting, external validity, internal validity (bias), internal validity (confounding) and power. For the current review, the item ‘power’ was removed. This was decided as none of the articles mention power, and the researchers decided to follow the scoring system used in a similar published review article (McDermott et al., 2013). Some questions were not applicable to all study designs, and the maximum score varied accordingly based on design type. The maximum score achievable for RCT – 27, repeated measures – 22, before and after – 22 and case studies − 17. O’Connor et al.’s. (2015) scoring was used to assess the Downs and Black scoring but modified to incorporate questions not applicable due to study design and removal of power, with excellent (25–27), good (19–24), fair (14–18) and poor (<14). See supplementary file for modified Downs and Black checklist used in this review.

CASP (2018) assessed qualitative studies and consisted of ten questions with no scoring system. Both tools assessed mixed-methods studies. Once again, discrepancies were discussed between the two reviewers and unresolved discrepancies resolved by the third reviewer. See Supplementary files for the CASP checklist used.

### Data extraction

The reviewers independently completed data extraction using a data extraction form created by the first author. Data extracted included- Title, Authors, Date, Contact detail, Country of origin, Abstract, Study design, Aims, Eligibility Criteria, Recruitment, Sample size and Characteristics, Setting, Intervention, Facilitator, Characteristics of Intervention, Control, Outcomes, Measurement tools, Statistical techniques, Findings and Limitations. Any discrepancies were resolved through discussion between the reviewers.

### Analysis

Initial descriptions of the findings were produced before concept mapping was used to highlight relationships between included studies. The authors went through the extracted data and gave a keyword that represented each finding, such as non-verbal, group membership, reminiscence. The keywords then formed themes; each keyword was written on sticky notes with the reference for the study. The authors explored the assigned theme, and any keywords that covered the same theme but were worded differently were reviewed and assigned a more fitting theme name. Related themes on the sticky notes were then grouped to identify relationships between studies. An overarching theme was created that represented the clusters of subthemes; these overarching themes were then used as the subheadings for the findings. Analysis was completed using A3 paper and sticky notes to visually move the themes around and see the relationships emerging between each study. It was decided that all studies would be included in the analysis despite methodology score to highlight the quality of studies.

### Findings

#### Narrative synthesis element two: Developing a preliminary synthesis

**Figure 2.**
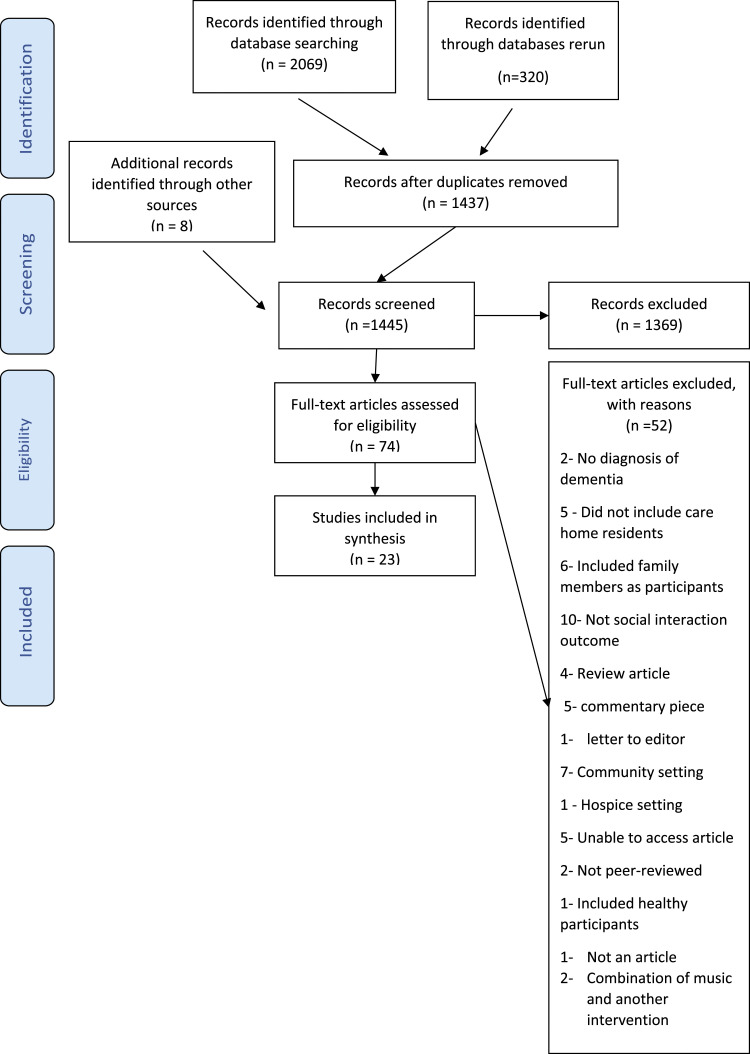
Included and excluded articles including reasons for exclusion.

The database searches retrieved 2069 results, and a further eight articles were retrieved during the hand search. In the literature search re-run, an additional 320 articles were retrieved. During the screening stage, 102 discrepancies were recorded at the title and abstract level, 91 were resolved after discussions, and 12 with the third reviewer. Seventeen discrepancies were recorded at the full-text level, 15 were resolved after discussions, and two with the third reviewer. A total of 23 articles were included in the synthesis (Figure 2). Eight of the included articles used quantitative methods, which consisted of one controlled trial, four repeated measures and three before and after study designs. There was one mixed-method article consisting of an RCT and observational study design. The remaining 14 articles were qualitative methods consisting of interviews and observations. See supplementary files for table presenting study characteristics of included studies.

#### *Narrative synthesis element 4:* Assessing the robustness of the synthesis

##### Methodology quality

During the methodology quality check, 50 discrepancies were recorded on the CASPS and 69 discrepancies on the Downs and Black tool. All discrepancies were resolved with the two reviewers.

In the Downs and Black assessment tool, Engström et al. (2011a), Hsu et al. (2015) and Raglio et al. (2008) scored ‘good’. The remaining quantitative studies scored between ‘poor’ and ‘fair’. Clearly described aims, outcomes and participant characteristics were reported across all studies. All applicable studies reported adjusting for the difference in lengths of follow up. Although some (42.9%) partially described confounding variables’, Raglio et al. (2008) was the only applicable study to fully describe or adjust for confounding variables. Adverse effects were only reported by Hsu et al. (2015). From most articles, it was difficult to determine the sample and settings representativeness due to poor reporting of eligibility criteria and recruitment strategy. The blinding of the outcome measures was only reported by Raglio et al. (2008). However, the nature of communication and observational data collection can make blinding the researcher challenging. All studies bar two documented adequate intervention reporting (Hammar et al., 2012; Sambandham & Schirm, 1995). Sambandham and Schirm (1995) was the only study not to report the main findings adequately. It was difficult to determine whether outcome measures were accurate in three studies (Engstrom et al., 2011a; Lesta & Petocz, 2006; Pollack & Namazi, 1992) and the appropriateness of statistical analysis in three studies (Hammar et al., 2012; Raglio et al., 2008; Sambandham & Schirm, 1995). Raglio et al. (2008) did not use standardised randomisation.

Most of the qualitative studies scored reasonably high on CASP. Kydd (2001) and Ridder & Aldridge (2005) scored the lowest with four out of ten. All studies were scored to have appropriate methodologies. Hsu et al. (2015) was the only study to report the recruitment strategy adequately. Clear aims were reported in 80% of the studies. Nine out of the 15 studies reported researcher-participant relationships inadequately. All studies bar Kydd (2001) reported clear findings. The reporting of methodology was insufficient in several studies, making it difficult to determine the appropriateness, including study design (Götell et al., 2009; Hammar et al., 2010; Hsu et al.,2015; Kydd, 2001), data collection (Dassa & Amir, 2014; Kuot et al., 2020; Kydd, 2001; Ridder & Gummesen, 2015), ethics (Hammar et al., 2011a; Kydd, 2001; Ridder & Aldridge, 2005) and data analysis (Dassa & Amir, 2014; Kydd, 2001; Ridder & Aldridge, 2005). See supplementary file for the table presenting the studies’ methodology quality.

### Intervention characteristics

Eight articles investigated music therapy (Dassa & Amir, 2014; Hsu et al., 2015; Kydd, 2001; Lesta & Petocz, 2006; Pollack & Namazi, 1992; Raglio et al., 2008; Ridder & Aldridge, 2005; Ridder & Gummesen, 2015). Music activities were explored in 15 studies that were facilitated by carers (Engström et al., 2011a; 2011b; Götell et al., 2002, 2009, 2012; Hammar et al. 2010, 2011a, 2011b, 2012; Kuot et al., 2020; Sambandham & Schirm, 1995; Swall et al., 2020), musicians (Campbell et al., 2017; Clare et al., 2019) or the researcher (Olderog Millard & Smith, 1989).

The layout and context of music activities varied across studies. Nine articles investigated music therapeutic care (MTC); however, only five unique separate studies and datasets were explored across the nine articles (Engström et al., 2011a, 2011b; Götell et al., 2002, 2009,2012; Hammar et al., 2010, 2011a, 2011b, 2012). The MTC articles are all related, with papers published from one research team at the same university, with several of the articles being part of a doctoral research project. MTC consists of carers singing or humming, on an individual basis, whilst completing tasks, including personal morning care, mealtimes and personal transfer. Background music was also explored in some studies. Swall et al. (2020) explored incorporating music into daily routines. One study explored personalised music listening (Kuot et al., 2020)

The additional fours studies explored group music sessions consisting of singing, music instrument playing or both. In Olderog Millard and Smith (1989), popular songs were sung whilst the researcher played the guitar. No details were reported in Sambandham and Schirm (1995), other than the intervention being facilitated by care staff. In Campbell et al. (2017), musicians provided music instrument improvisation sessions. Live music sessions were investigated in Clare et al. (2019), consisting of musicians and residents singing and playing instruments in group and individual interactions.

The music therapy studies were less inconsistent; they consisted of group sessions (Dassa & Amir, 2014; Lesta & Petocz, 2006), individual sessions (Pollack & Namazi, 1992; Raglio et al., 2008; Ridder & Aldridge, 2005; Ridder & Gummesen, 2015) or both (Kydd, 2001), facilitated by a licenced music therapist. Hsu et al. (2015) had the additional element of indirect staff skill-sharing. Studies used a range of music therapeutic skills, including turn-taking, imitation, holding, containing, validation, empathy and modelling. The residents’ behaviours and needs mainly guided the sessions and therefore varied in activities, but singing, music listening, music playing and reminiscence were all reported across studies.

### Participant characteristics

*Music activities*. Campbell et al. (2017) and Swall et al. (2020) did not report any participants’ characteristics, including the residents’ sample size. Across the remaining studies, 97 residents were recruited aged 67–98. The studies that reported gender consisted of 27 females and 13 males. Age was not reported for four studies (Götell et al., 2012; Hammar et al., 2010, 2011a, 2011b) and gender was not reported in Götell et al. (2012), Kuot et al., 2020 and Sambandham and Schirm (1995). The dementia severity measures varied; however, all participants were reported to have moderate to severe dementia.

Eight of the studies included staff participants. Sambandham and Schirm (1995) did not report the sample size or characteristics. From the remaining seven studies, 79 participants aged between 20 and 63 were recruited. The studies that reported gender consisted of 53 females and two males. The name of staff job roles varied between studies, but all were reported to either be licensed nurses or carers. Engström et al. (2011a) and Kuot et al., (2020) did not report age, gender, or job roles. Campbell et al. (2017) only reported interview numbers, which consisted of a music therapist, musicians, members of the organisation team, activity workers and a care home manager; age and gender were not reported.

*Music therapy*. Across the studies, 98 residents participated aged between 64 and 97, consisting of 79 females and 19 males. Kydd (2001) did not report dementia severity; the other studies reported dementia severity between moderate to severe. One study consisted of ten carers aged between 21 and 60 and consisted of seven females and three males (Hsu et al., 2001).

### Narrative synthesis element 3: Exploring the relationships within and between the studies

Four key themes emerged: social interactions prior to the intervention, Staff experience, Residents’ social interactions during and post-intervention and Mechanisms. Many of these themes emerged from both music activities and therapy despite the difference in interventions. See supplementary file for the table presenting a summary of study findings.

### Social interactions prior to the intervention

Residents’ social interactions prior to the intervention were explored mainly in MTC (Engström et al., 2011a, 2011b; Götell et al., 2002, 2009, 2012; Hammar et al., 2010, 2011a, 2011b, 2012); and music therapy studies (Kydd, 2001; Ridder & Aldridge, 2005; Ridder & Gummesen, 2015). During the MTC and music therapy studies, it was challenging to engage residents in positive interactions prior to the intervention, whether with staff, other residents or family members. Many were isolated from social contact, leading to other issues such as depression.

In Kydd (2001), the participant remained in his room apart from at mealtimes and did not engage in communal activities or interactions. He could not follow the group format when attending only group music therapy, including selecting irrelevant songs and leaving before the end. In many studies, verbal communication was difficult for residents to use and comprehend. Some residents’ verbal language consisted of repeating a single word or phrase (Ridder & Aldridge, 2005; Ridder & Gummesen, 2015). However, many of the participants across the studies were able to comprehend and use non-verbal communication. Ridder and Aldridge’s (2005) participant was the only individual reported to have a disinterest in music prior to the session.

In the MTC studies (Engström et al., 2011a, 2011b; Götell et al., 2002, 2009, 2012; Hammar et al., 2010, 2011a, 2011b, 2012), carers aimed to verbally narrate their actions, despite residents’ inability to comprehend, leading to them repeating instructions. Carers used strong, energetic tones to portray warmth and enthusiasm; however, residents were confused and frightened, which was reflected in their speech. Residents’ verbal communication was limited, and when used, was generally a weak flat tone with monosyllables, incoherent speech and out of context sentences. Their primary forms of communication were muteness and ‘resistance to care behaviours’. Carers referred to residents being physically present in the room but not mentally, making communication difficult and one-sided. However, researchers also reported that carers provided little opportunity for residents to interact actively. Both parties worked at different paces, leaving carers feeling lonely and powerless.

### Residents’ social interaction during and post-intervention

The studies, despite intervention type, all reported changes in residents’ interactions. However, the degree of change varied across the studies. Many reported increased sociable verbal and non-verbal communication, whilst observing a decrease in unsociable verbal and non-verbal communication (Clare et al., 2019; Engstrom et al., 2011a, 2011b, Götell et al., 2002, 2009, 2012; Hammar et al., 2010, 2011b, 2011a; Kuot et al., 2020; Kydd, 2001; Olderog Millard & Smith, 1989; Pollack & Namazi, 1992; Raglio et al., 2008; Ridder & Gummesen, 2015; Sambandham & Schirm, 1995; Swall et al., 2020). Residents vocally participated more, and speech became more fluid and coherent. Verbal communication remained difficult for many residents; however, singing and music allowed them to express themselves. Even though residents were more capable of using non-verbal communication before the intervention, the studies reported increased eye contact, smiling and body movements. These improvements were reported to remain after the intervention. Participants were more sociable and interactive at other times of the day (Hsu et al., 2015; Kydd, 2001; Pollack & Namazi, 1992 Sambandham & Schirm, 1995), in turn, reducing social isolation.

The increase in verbal and non-verbal communication was not significant across all studies. In Hammar et al. (2012), there was minimal change between usual care and MTC. However, argumentative, rejecting and ‘verbalising negative affection’ were reported to diminish. The only recorded verbal communication used by the resident was humming in one session. In Lesta and Petocz (2006), the findings were more inconsistent with all outcomes, both sociable and non-sociable, except reminiscence significantly improved overall. The post hoc tests highlighted that most outcomes bar mumbling and wandering alone significantly improved during therapy compared to pre-therapy baseline scores. However, mumbling, wandering alone, eye contact and talking were the only outcome to improve significantly post-therapy compared to during therapy. Additionally, significant improvements from pre to post-intervention was only reported in mumbling, sitting alone, walking and sitting with others. Some of these outcomes, such as sitting alone, may not demonstrate an improvement in social interactions; there may have been other factors affecting the outcomes, such as availability of seating. In Hsu et al. (2015), there was no significant difference in personal enhancers between conditions. Despite this, carers reported improvements in residents’ communication skills post-therapy. The use of music was not positive for all residents; in particular, one participant in Kuot et al. (2020) felt she was being buried alive when certain songs were played.

Music therapeutic care orientated residents to the present and improved alertness, which may explain the improvements in fluency and coherency of speech as well as verbal communication comprehension (Engstrom et al., 2011a, 2011b, Götell et al., 2002, 2009, 2012; Hammar et al., 2010, 2011b, 2011a). In both music therapy and music activities studies, an increase in laughter and humour between residents, staff, and other members strengthened connections and allowed residents to express positive emotions (Clare et al., 2019; Campbell et al., 2017; Engstrom et al., 2011a; Götell et al., 2009, 2012; Hammar et al., 2010, 2011a, 2011b; Kuot et al., 2021; Ridder & Gummesen, 2015; Swall et al., 2020). In Dassa and Amir (2014), residents provided singing advice to other members, which aided in developing a collective identity. Music helped develop a sense of togetherness that facilitated socialisation and cooperation (Olderog Millard & Smith, 1989; Swall et al., 2020). In all music intervention types, residents were observed singing, even when verbal language was severely impaired (Götell et al., 2002, 2012; Hammar et al., 2010, 2011a, 2011b; Kuot et al., 2021; Lesta & Petocz, 2006; Olderog Millard & Smith, 1989; Ridder & Gummesen, 2015; Swall et al., 2020). The ability to sing and remember lyrics improved self-esteem and confidence, encouraging communication through music (Dassa & Amir, 2014). In Swall et al. (2020), some participants were unable to sing; however, they were able to have reciprocal communication without the need for words through movement and shared music experiences. Residents who display agitated behaviour, even when interacted with verbally, relaxed and calmed when music was introduced, providing a time and space for residents and facilitators to communicate through music and non-verbal communication (Hsu et al., 2015; Ridder & Aldridge, 2005; Ridder & Gummesen, 2015). Ridder and Aldridge’s (2005) participant was the only individual to have little reaction to the intervention due to her impairments; however, touch was still observed as a form of communication that aided the development of a relationship between the therapist and resident.

### Staff experience

Some studies consisted of care staff as facilitators or participating members (Campbell et al., 2017; Clare et al., 2019; Engstrom et al., 2011a; 2011b; Götell et al., 2002, 2009, 2012; Hammar et al., 2010, 2011a, 2011b; Hsu et al., 2015; Swall et al., 2020). Staff were made aware of small non-verbal signals, which had previously been missed or misinterpreted (Campbell et al., 2017; Clare et al., 2019). As a result, staff could alter their communication style to accommodate the resident. Through reminiscence and meaningful interactions, staff gained greater insight into residents’ lives and personalities, allowing them to see the identity behind the diagnosis (Campbell et al., 2017; Clare et al., 2019; Hsu et al., 2015). In the MTC studies, staff reported relationships and interactions as one-sided and limited, if not completely non-existent, during usual care (Engstrom et al., 2011a, 2011b; Götell et al., 2002, 2009, 2012; Hammar et al., 2010, 2011b, 2011a). Through music, a mutual understanding was developed, with staff reporting more reciprocal relationships, where both parties were actively engaging. The reciprocal nature of interactions during music was also explored in Swall et al. (2020). Improvements in communication skills were observed in both residents and staff. The care staff in Swall et al. (2020) became more aware of interactions, allowing them to adapt them to be more person-centred and meaningful. Staff felt music allowed them to provide instructions more gently and respectfully. In the MTC studies, staff increased their use of non-verbal communication and reduced verbal language (Engstrom et al., 2011a, 2011b Götell et al., 2002, 2009, 2012; Hammar et al., 2010, 2011b, 2011a). The researchers highlighted the paradoxical findings but suggested fewer instructions were required due to improved awareness and interactions. Therefore, tasks were completed more effectively without the need for intense narrative. The addition of the staff skill-sharing in Hsu et al. (2015) allowed staff to include effective music interaction skills into daily practices, ensuring that benefits remained post sessions.

### Mechanisms of music for facilitating social interactions

**Figure 3.**
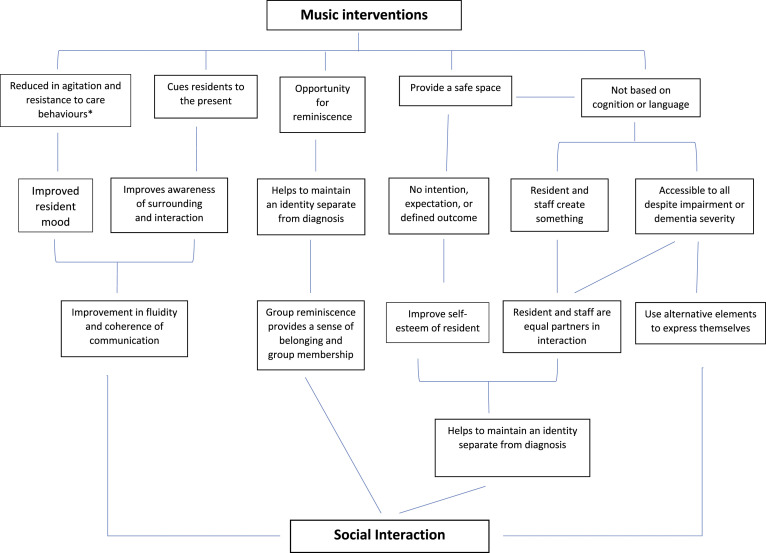
The model highlights the authors interpretation of the result presented in the narrative synthesis to present a model that highlights the potential mechanisms to explain how care home music interventions can facilitate social interaction. The lines highlight the connection the authors have made between the mechanisms. Many of the mechanisms were highlighted in both music therapy and music activities. *Reduction in resistance to care was seen in the studies where music was used during care tasks, Reduction in agitation was seen in all music intervention.

The articles highlighted potential mechanisms of music that could facilitate social interactions, which were observed in both music therapy and music activities (Figure 3). The interventions were not based on cognition or verbal language, which allowed residents to engage without their impairments hindering their experience. Adaptability allowed interventions to be tailored to residents’ needs and abilities, making them inclusive for all, despite dementia severity. Music was highlighted as an alternative communication form for residents to express themselves when verbal language deteriorates. The use of musical turn-taking and mirroring allowed residents to be actively involved in interactions and build relationships (Clare et al., 2019; Ridder & Gummesen, 2015). Improvisation allowed participants to create something together as equal partners, strengthening connections (Campbell et al., 2017; Clare et al., 2019; Kydd, 2001; Ridder & Gummesen, 2015). Sessions provided a safe space for residents, staff and/or facilitators to communicate without intentions, expectations or defined outcomes. Staff learnt it was acceptable to allow interactions to occur naturally. During periods of silence, staff discovered interactions were still occurring, with residents psychological, social and emotional needs still being met (Clare et al., 2019; Campbell et al., 2017). A safe space was crucial for those residents usually uncomfortable with communicating. In Campbell et al. (2017), the musicians were attuned to the residents’ vulnerability, which enabled them to alter the environment and interactions, such as being aware of more subtle communication cues, as well as knowing when residents needed stillness and silence.

Music acted as a cue to orientate to the present, with residents becoming more aware of their surroundings, and improving interaction’s fluidity and coherence. A prominent theme was reminiscence. Music from the residents’ earlier years evoked personal memories that allowed for discussions around life stories (Hsu et al., 2015; Campbell et al., 2017; Dassa & Amir, 2014; Götell et al., 2002; kuot et al., 2020; Lesta & Petocz, 2006; Pollack & Namazi, 1992; Sambandham & Schirm, 1995; Swall et al., 2020). The music in Dassa and Amir (2014) revolved around cultural or historical events, which evoked patriotism, leading to the development of a sense of belonging.

The authors developed a model to visually highlight their interpretation of the analysis of findings from the original papers. The model highlights the potential mechanisms that could explain how care home musical intervention facilitate social interactions. Although the aims and context of music therapy and music activities varied, the mechanisms were present in both intervention types to some degree.

## Discussion

Twenty-three papers were included in the final synthesis, which consisted of music activities and music therapy. Although the music interventions differed in their approaches and purpose, overall studies reported increased residents’ sociable verbal and non-verbal communication and decreased unsociable communication.

In the findings, elements two, three and four of the narrative synthesis was explored. Once the data was extracted, an initial synthesis of the findings (Element 2) was completed to gain an understanding of the effects of the interventions. The initial synthesis was then expanded to identify the relationships within and between the studies (Element 3). Element three allowed the researchers to explore why and how interventions facilitated social interactions and the barriers and enablers that may have impacted the effectiveness. Within the result section, the researchers also assessed the methodology quality of the included studies, which contributed to the first stage of assessing the robustness of the synthesis (Element 4). In the discussion, the researchers will further explore the relationships between the included studies (Element 3) with reference to other published studies to contextualise the findings. The robustness of synthesis (Element 4) will be assessed further by exploring the limitations of the included studies and the limitations of the review itself.

Music therapy and music activities explored in this review highlighted similar mechanisms to explain how care home music interventions facilitate social interactions, including providing an opportunity to reminisce, providing a safe space and being inclusive for all. Although the findings have highlighted similar mechanisms, it has been argued that music activities cannot substitute music therapy. McDermott et al. (2018) argues that music interventions rely on the delivery of the intervention and the facilitator, as much as the intervention content. As music activities are generally provided by staff, musicians or volunteers, they would not have the same level of training as music therapists and therefore may struggle to provide high-quality interventions. Additionally, the aims of music therapy and music activities vary; therefore, the intervention best suited for the resident will be dependent on their needs. Although music activities cannot substitute music therapy, there is a need for them within care homes, and this review highlights their benefits. The findings from this review suggests a vast range of activities can be facilitated by either care staff or musicians that provide similar benefits to music therapy to facilitating social interactions. These findings are promising for UK care homes because access to music therapy is limited by funding and therapist availability (Schneider, 2018). Some form of music activity could be accessible to all care homes to help facilitate social interactions. However, a recent report suggests that only 5% of residents have access to quality arts and music interventions (Bamford & Bowell, 2018).

The findings suggest music can improve social isolation, alertness, moods, connecting with others, self-worth, maintaining an identity and ability to express themselves. Many UK residents could be potentially missing out on these benefits, leading to lower quality of life and care and increased behavioural and psychological symptoms of dementia. Lack of staff time and workload pressures have been highlighted as limiting factors in delivering music activities (Ekra & Dale, 2020; Stanyon et al., 2016; Sung et al., 2011). However, the MTC studies highlighted that staff-led musical interactions could be provided without additional time or workload burdens and without compensating on the quality of physical care. Staff also reported that care tasks were completed more efficiently, quicker and calmer because residents increased awareness and communication comprehension.

The studies highlighted music accessibility to all individuals with dementia, although factors such as dementia severity and residents’ needs may vary engagement. Interventions were all adaptable to be inclusive, allowing different abilities to actively engage with the intervention, either on their own or with other residents at the same time. The slightest engagement, such as eye contact or touch, were reported beneficial. Social interactions are vital for humans, even when they may be unable to equally reciprocate the communication (Moyle et al., 2013). The findings also recognised Kitwood’s (1998) theory of personhood. Interventions aimed to meet residents’ fundamental needs beyond physical health and safety, leading to improved communication, behaviour and well-being. Although the studies in this review imply that residents’ needs were considered to guide the intervention, Hackett et al. (2021) argues that many music interventions lack individual goals. Instead, a single goal is assigned to all individuals in the intervention despite their differences, which could affect the effectiveness of facilitating social interactions.

The findings supported McDermott’s et al. (2014) psychosocial model that music can facilitate social interactions by allowing individuals to maintain their identity and connect with others. Residents used music to connect, with staff discovering the identity behind the diagnosis. In group sessions, residents connected by discussing joint experiences such as cultural and historical events, creating a sense of belonging. Although some studies highlighted group membership, participants’ culture, traditions and/or country of research may impact the sense of belonging. In Dassa and Amir (2014), although the participants had diverse cultural backgrounds, all grew up in Israel and may have had similar experiences. In larger diverse countries such as the USA or the UK, residents may have vastly different experiences; thus, reminiscence may not create a sense of group membership. If some residents’ country of origin differed from where they currently live, the opposite effect might be observed if the activity focuses solely on traditional music. When Sonas, an intervention developed and implemented in Ireland, was researched in the UK, the Irish songs and poetry were not altered. The British participants with dementia had no familiarity with the songs (Hutson et al., 2014). The lack of connection with the songs and poetry may explain the lack of clear benefits that had previously been found in Ireland. The Sonas’ study suggests that music should be personalised to the individual and in line with their culture and group identity.

The discussion of barriers to successful implementation was limited among studies. Campbell et al. (2017) argued that contextual, structural and organisational considerations could impact music interventions in care homes. The care staffs’ attitudes are a primary necessity for successful implementation. The ability to be flexible and adaptable to the residents’ needs, moods and health were also necessary (Hsu et al., 2015; Kuot et al., 2020). In Kuot et al., (2020), between 4 and 10% of sessions were missed due to sleeping or unwillingness to attend; flexibility in start time could improve attendance rates. Additional time from care staff may be required to provide support when dementia symptoms make accessing music interventions challenging, such as forgetting about sessions. As mentioned previously, staff are limited by available time, making accessing training and implementing interventions difficult (Boersma et al., 2015; Hsu et al., 2015; Keenan et al., 2020; Lawrence et al., 2012). In Hsu et al. (2015), despite staffs’ positive attitudes, the skill-sharing element was challenging to implement due to staff shortage and time pressures. High turnover in staff can also impact implementation, as new staff require training (Kuot et al., 2020). In the studies, staff had little singing experience and may have felt embarrassed; more training in singing and using music could be a positive enabler to ensure successfully implemented into practice. (Götell et al., 2009, 2012).

### Narrative synthesis element four: Assessment of the robustness of the synthesis

#### Limitations of studies

A significant limitation of the studies was limited follow-up data collection. Most studies only collected data during or immediately after the music intervention. The lack of longitudinal studies indicates difficulties in determining the longer-term effects of music on social interactions, which has also been highlighted by previous systematic reviews (Van der Steen et al., 2018; Vink et al., 2003). However, Hackett et al. (2021) argues that the mechanisms of music interventions would not achieve a longitudinal effect after the intervention ended. Instead, music should be provided in daily dosages to maintain regular benefits.

In addition, interview studies omitted collecting data from residents. Whilst it can be challenging to collect data directly from individuals with dementia, there is a missed opportunity to gain insight from the residents’ perspective. Previous research highlights differences in perception of residents’ well-being, care and health between residents and care staff (Buckley et al., 2012; Griffiths et al., 2020). Therefore, the interview studies portray a carers perception of social interaction in care homes rather than an accurate representation of residents’ interactions.

The first author was involved in the study and analysis stage for many studies; this could lead to a ‘Researcher effect’ with preconceptions influencing their analysis. Discussions with other researchers were used to overcome this issue. Similarly, in the music therapy studies, many therapists were also the researcher, which could have impacted the therapist’s behaviour during sessions and the researcher’s behaviour during analysis. Dassa and Amir (2014) reported this issue when the roles of music therapist and researcher conflicted. Similarly, Lesta and Petocz (2006) reported that observers became quasi group members when issues arose with residents, leading to bias issues and lack of internal validity and reliability.

### Limitation of review

The included articles were all written in English from peer-reviewed journals. As a result, articles may have been excluded that could provide greater insight into facilitating interactions through music. The decision to omit non-peer-reviewed articles could have led to this review displaying publication bias. However, the authors wanted to create a transparent and rigorous review. Due to limited resources and the review being required for a time-limited project, it was decided only to include peer-review journal papers. The quality of this review relies on the quality of the studies’ methodology. It was decided all studies would be included to highlight the quality of current music interventions; however, due to the lower methodology quality, the findings should be interpreted with caution. The limited reporting of music intervention in some studies made it difficult to highlight relationships between studies and determine potential mechanisms for how music interventions facilitate social interactions.

### Practical implications

This systematic review has highlighted that introducing music activities into care homes could benefit most residents, despite the severity of their dementia. The interventions could improve verbal and non-verbal communication, provide insight to staff on the person behind the diagnosis, and improve social participation and task efficacy. Furthermore, the staff-led interventions highlighted the plausibility for staff with no music training to implement music skills. The most appropriate intervention will vary between care homes, and several factors influence the appropriateness, including finances, availability of facilitator, residents’ preferences, needs and abilities. In the staff-led interventions, many lacked the confidence and knowledge to implement music interventions. If interventions are not high quality, they could lack efficacy or be harmful (McDermott et al., 2018). Therefore, there is a need to develop an accessible and standardised manual to ensure that implemented staff-led music interventions are of high quality. Alternatively, a combination of music therapy and staff skill-sharing, as present in Hsu et al. (2015), could enable staff to incorporate music into care with a music therapist’s support to ensure the benefits continue outside of the music therapy sessions. The combination of music therapy and staff skill-sharing has been explored in McDermott et al. (2018). Residents can continue to communicate when verbal communication deteriorates. However, care professionals need to recognise residents’ alternative communication behaviours and respond appropriately.

### Future research

The included articles had low to medium methodology quality. Therefore, future research should focus on improving methodology quality in music intervention studies. In particular, reporting the interventions’ process in more detail will ensure research can be replicated and successfully implemented. Although briefly mentioned, future research should consider the barriers and enablers to successfully implementing care home music interventions. The findings of MTC highlighted the potential of singing during care to improve interactions; however, as the articles were produced by the same research team and consisted of five studies, the data should be considered with caution. Future research into MTC is required to ensure the findings are replicable. Finally, residents were passive participants in the research process in the included studies. Future research that invites residents to attend interviews could produce valuable insight into their experience.

## Conclusion

There is evidence that both music therapy and music activities can facilitate social interactions for people with dementia in care homes. Residents displayed increased verbal and non-verbal communication and interacted more with carers, music therapists, session leaders and other residents. Benefits were not exclusive to those with sufficient verbal communication abilities; many participants with language impairments were observed singing. For those with no language, the sessions were still inclusive by using non-verbal communication, suggesting that music can benefit a wide range of residents with dementia. Finally, the findings only highlight increased communication during or immediately after the music intervention; as previous research highlights, regular, continuous dosages of music rather than a one-off intervention would ensure benefits remain. Quantitative studies provided inconsistent findings, with many exploring singing describing social behaviour in an unconventional way, such as ‘walking together’, rather than communication and interaction outcome. This may have been due to the subjective nature of interactions or the lack of tested, standardised outcome measures for social interactions. Standardised outcome measures were used in some studies, however, many had to be modified, and the verbal and non-verbal interaction scale had not been tested at the point of use (Williams et al., 2017). This review highlights a potential need to develop quantitative outcome measures that are more fit to measure social interactions in individuals with dementia.

## Supplemental Material

Supplemental Material – Analysing the use of music to facilitate social interaction in care home residents with dementia: Narrative synthesis systematic review Supplemental Material for Analysing the use of music to facilitate social interaction in care home residents with dementia: Narrative synthesis systematic review by Bryony Waters, Martin Orrell, Lídia Sousa and Orii McDermott in Dementia
