# Supplementary material for: Analysing the use of music to facilitate social interaction in care home residents with dementia: Narrative synthesis systematic review
Source: Dementia (London). 2022 May 20;21(6):2072–94. doi: 10.1177/14713012221100625 (PMC12064862; doi:10.1177/14713012221100625)
Supplement: Supplemental Material – Analysing the use of music to facilitate social interaction in care home residents with dementia: Narrative synthesis systematic review [file sj-pdf-1-dem-10.1177_14713012221100625.pdf]

Title and description for supplementary files

- Table presenting the study outcomes, summary of findings and methodology quality

  The table in this supplementary file provides the study outcomes and measures, summary of findings and the results of the methodology quality check.

- Table presenting study characteristics.
  The table in this supplementary file provides the study characteristics including author, year of publication, intervention, sample size and characteristic, study design and length of study for the included studies.

- Downs and Black methodology quality decisions
  The table in this supplementary file presents the two reviewers' final decision for each question of the Downs and Black methodology quality tool.

- CASP methodology quality decisions
  The table in this supplementary file presents the two reviewer's final decision for each question of the CASP methodology quality tool.

Table presents the outcomes each study explored, the data collection methods used, a summary of the key findings and the methodology quality check findings

**Author (Year)**	**Outcomes (data collection methods)**	**Summary of findings**	**Methodology Quality Check**
		**Qualitative studies**	
**Campbell et al. (2017)**	Participation Experience (Interviews & Diary entries)	• Allowed residents to make choices and develop relationships<br>• Improved awareness<br>• Staff gained insight and understanding of the resident and their abilities<br>• Staff learnt different types of communication styles. Learnt to allow interactions to happen naturally, and it is okay to be together in silence<br>• Residents used music to express themselves	CASP<br>9 /10<br>Care home and participant recruitment not reported

**Clare et al. (2019**	Nature and range of interaction changes in communication skills (Video Observation)	• Live music facilitates both verbal and no-verbal communication<br>• Communications is a network of subtle and complex interactions with no linear form, creating a multisensory environment<br>• Interactions required the initial communicative action to be noticed by another member and for them to respond<br>• If communicative action were missed, residents cease communication attempt	CASP<br>9/10<br>Care home and participant recruitment not mentioned
**Dassa & Amir (2014)**	Connection between musical features and conversation topics (Video Observation)	• Two types of conversations 1) relating to the context of the song 2) relating to the activity of group singing<br>• Songs evoked memories of their country or personal events<br>• Members provided advice on singing to other members, Provided a sense of belonging.<br>• Paid compliments to other members.	CASP<br>7 /10<br>Recruitment not reported<br>Not sufficient reporting of data collection methods and data analysis methods
**Götell et al.(2002)**	Verbal communication (Video Observation)	• Care staff reduced verbal communication but increased nonverbal<br>• Despite decreased verbal communication, residents understanding increased.<br>• Silenced residents became more talkative<br>• Residents verbal communication became clearer and more mutual.<br>• MTC interactions were mutual.<br>• Reminiscence	CASP<br>8 /10<br>unable to determine clear aims<br>Recruitment strategy unclear
**Götell et al. (2009)**	Communicating emotions and mood (Video Observation)	• Control condition- cares conveyed friendliness, warmth, and engagement through an energetic voice<br>• Control condition- Residents had weak flat, monotone voices. Communication was fragmented and short<br>• Intervention condition- residents more vocal, with both residents and staff, speaking with a warm tone<br>• Interactions were more equal<br>• Verbal communication was not always necessary to express emotions.<br>• Residents playfully responded to carers<br>• Both parties connected physically and emotionally with mutual appreciation expressed.	CASP<br>6 /10<br>no clear aim<br>Unable to determine appropriate study design or recruitment<br>Researcher and participant relationship not reported

**Götell et al. (2012)**	Communication (Interview)	• Enhanced mutual verbal and non-verbal communication<br>• Singing improved the efficacy and ease of a task<br>• Residents had increased awareness, making communication easier<br>• Both parties expressed enhanced positive moods and emotions<br>• Increased humour, with residents making jokes and laughing<br>• Residents' language and speech improved	CASP 8/ 10. Appropriateness of recruitment strategy undetermined relationship between researcher and participants undetermined
**Hammar et al. (2011b)**	Carer's experience of communion (Focus group)	• Control condition- communication was difficult or impossible<br>• Residents were physically in the room but not mentally present<br>• Carers felt alone in the task and communication.<br>• Residents did attempt to show love through touch<br>• Intervention condition- communication was more mutual, with residents more aware and present.<br>• Improvement in residents' speech	CASP 9 /10 Appropriateness of recruitment strategy undetermined
**Hammar et al. (2011a)**	Communication (Video observation)	• Control condition- carers did not invite residents to participate in the communication<br>• Little eye contact from either party<br>• Carers and residents attempted to interact at different paces.<br>• In the intervention increase in eye contact was reported, and residents could actively engage more as both parties attempted to interact at the same pace.	CASP 8/10 Insufficient reporting of the recruitment strategy and ethics
**Hammar et al. (2010)**	Verbal and non-verbal communication (Video Observation)	• Control condition- carers used non-verbal communication to act out tasks but rarely invited the residents to join in the task or interaction.<br>• Little eye contact from either party<br>• Carers and residents worked at different paces, residents were slower, and carers regularly interrupt<br>• Intervention condition- carers invited residents to communicate more<br>• Increased Non-verbal communication elements became the primary form of communication<br>• Eye contact and humour increased	CASP 7/ 10 Unable to determine appropriateness of study design and recruitment strategy is appropriate. No reporting of researchers and participants relationship
**Ridder & Gummesen (2015)**	Communication (Video recording, Therapist log, Pre-assessment reports, Session transcripts)	• Resident struggled to understand verbal communication, had limited verbal language, repeated a single phrase<br>• He did understand and use non-verbal communication<br>• He used the drum app on the iPad to explore sounds and engage in rhymical playing<br>• Singing, turn-taking, and mirroring were used to connect	CASP 7 /10 Unable to determine appropriateness of

	.	• He used different tones of voice and gestures to express himself<br>• The joint sing helped develop a symbolic relationship leading to the resident feeling safe.	recruitment strategy and data collection<br>No reporting of researcher and participant relationship
**Kydd (2001) (11)**	Social interaction<br>(Direct observation)	• At the beginning, he was reluctant to play his banjo as he felt he was not as good as he used to be<br>• He struggled to follow group music session rules and would leave early<br>• As sessions progressed, participation increased with him following rules<br>• He became more sociable at other times of the day by increasing interactions with others<br>• During the sessions, he would reminisce and performed for the other residents.	CASP<br>4 /10.<br>Unable to determine appropriateness of recruitment strategy<br>No reporting of researcher and participants relationship<br>Insufficient reporting of ethics, data collection and clear findings
**Ridder & Aldridge (2005)**	Behavioural effects of music (Video recording, Modified Cohen-Mansfield agitation inventory (Cohen-Mansfield, 1986), Music therapist log)	• Challenging to engage the resident in positive interactions due to serve dementia<br>• Prior to sessions- isolated from social contact with staff and residents<br>• During sessions- She used touch to connect and pulled the therapist up from the sofa to walk with her. a sign of mutual understanding.<br>• She repeated a single word, and the use of the word increased<br>• The resident did not actively participate in sessions	CASP<br>4 /10<br>Unable to determine the aims, researcher and participant relationship, the appropriateness of study design, recruitment, ethics, and rigorousness of analysis
**Kuot et al. (2020)**	Behaviour, well-being, clinical management, culture, and social interaction (Focus groups)	• The intervention did not work for all residents.<br>• Music improved social interaction with staff and other residents<br>• Staff more playfulness with residents<br>• Reduction in resistance to care behaviour<br>• Music is a conversation point	CASP<br>7/10<br>Unable to determine the researcher participant relationships, the appropriateness of

			recruitment and data collection
**Swall et al. (2020)**	Feeling behaviours of residents and Carers (World café discussion)	• Intervention built bridges in a person-centred way to connect with residents<br>• Interactions more valuable, joyful, and meaningful, created opportunities to frame reciprocal communication<br>• The intervention promoted togetherness that facilitated socialisation and cooperation<br>• Promoted communication when language was no longer available.<br>• Singing was a respectful way to reach the residents.<br>• Music helped develop life stories for the resident and start discussions.	CASP<br>9/10<br>Unable to determine the appropriateness of recruitment
		**Mixed Methods**	
**Hsu et al. (2015)**	Carers and residents' interactions (Interview, Video observation, Dementia care mapping (Brooker & Surr, 2006), Neuropsychiatric Inventory (Cummings et al., 1994)	• Case study- musical vocal, bodily and facial expressions were used as emotional cues.<br>• The therapist's verbal expression agitated the resident, but soothing melodies were used as musical cues when verbal failed.<br>• The resident used eye contact and smiling in interactions<br>• The resident reminisced over playing the piano; this was fed back to staff use in routines.<br>• Quantitative study- Personal enhancers did not differ between the intervention and control group at 3 months (-24.08 95% CI [-97.47 to 49.32] P=0.294), 5 months (-28.08, 95% CI [-86.70 to 30.55], P=0.176) or 7 months (-18.83, 95% CI [-37.68 to 0.026], P=0.050).<br>• Carers reported improvement in mood, communication, self-expression, and agitation in the intervention group.<br>• Carers gained insight into residents' life history and Improved interaction, communication, and relationships.	CASP<br>8 /10<br>Unable to determine appropriateness of study design<br>No reporting of researcher and participant relationship<br>Downs & Black<br>19 /26
		**Quantitative studies**	
**Engstrom et al. (2011b)**	Social and unsocial communication behaviours (Video observation, Modified verbal & nonverbal communication scale (Williams, 2017))	• Significant improvement in 5 of the 9 sociable verbal communication behaviours includes coherent (t= -2.295, p<0.012) and relevant communication (t 975) = 2.726, p=0.009), humming (t (75) =3.032, p=0.004), singing (t (75) =4.43, p=0.0005), and responding to questions (t (75) =-5.17, p=0.0005).<br>• Residents' ability to respond to questions increased by 69% (baseline mean 1.53) (intervention mean 4.95).<br>• Unsociable non-verbal communication decreased by 80%, including cursing (t (75) = -2.14, p=0.037) and not responding to questions (t (75) = -4.92, P= 0.0005). Cursing only significant unsociable verbal outcomes to decrease out of 5 outcomes. Does not respond to questions only unsociable nonverbal outcome to significantly decrease out of 7 outcomes.	Downs and Black<br>9 /17

**Lesta & Petocz (2006)**	Mood, social behaviour, and non-social behaviour (Mood-behaviour assessment chart. (Author designed tool))	• Improved non-social and social behaviours<br>• All variables apart from reminiscence significantly improved- mumbling (0.002), sitting alone (,0.001), wandering along (p= 0.0011), walking with others (0.009), sitting with others (0.001), eye contact and smiling (p<0.001), talking (p<0.001).<br>• Social behaviours increased from pre to post-therapy, with eye contact and smiling (P=0.22), and talking (P=0.52) being non-significant and walking with others (P=0.030) and sitting with others (p= 0.005) being significant	Downs and Black 11 /21
**Olderog Millard & Smith (1989)**	Social behaviour, vocal participation (Behaviour mapping observation (Ittelson et al., 1970) Bell and Smith checklist (Bell & Smith, 1986))	• Significant difference between pre- and post-intervention in sitting (F (4,36) = 3.131, P= 0.0262) and walking together (F 9(4,36)- 3.129, P= 0.0263).<br>• Singing condition- significantly higher vocal participation (F (4,36) = 4.435, P=0.0051)<br>• Both conditions significant effect on residents' behaviour<br>• Smiling increased during the session, and touching other increased post-session.	Downs and Black 12 /21
**Pollack & Namazi (1992)**	Social and non-social behaviour, participation (Direct observation, Individual observation form, Behavioural checklist)	• 24% increase in social behaviours and 14% decrease in non-social behaviours post sessions. (x_2= 14.2, df=1, P<0.001)<br>• The most significant increase in social behaviour was non-verbal, which increased from 32% to 68%<br>• Increase in verbalisation and positive verbal feedback<br>• Reminiscence was present<br>• Residents' interactions becoming more fluent	Downs and Black 9 /19
**Hammar et al. (2012)**	Verbal and non-verbal interactions Facially expressed emotions (Video observation, Verbal, and nonverbal interaction scale (Williams 2017), Observed emotion rating scale (Lawton,2006))	• Eye contact decreased in the second baseline and intervention sessions<br>• The resident could not verbally interact, which stayed consent across sessions<br>• Resident hummed in one session<br>• carers interactions remained the same, but verbal communication decreased	Downs and Black 8 /20
**Engstrom et al. (2011a)**	Social and unsocial communication behaviour (Video observation,	• Sociable verbal and nonverbal communication increased by 23% from 209 observations (mean-26.1) to 258 (mean- 32.3)<br>• Unsociable verbal and non-verbal communication decreased by 80% from 30 (mean- 3.8) to 6 (mean-0.8)<br>• The Resident asked appropriate questions more frequently	Downs and Black 13/ 21

	Modified verbal, and nonverbal interaction scale (Williams, 2017))	• Increased smiling and laughing<br>• Residents' language was more coherent but still challenging to understand	
**Sambandham & Schirm (1995)**	Communication, social skills, capacity to reminisce (Glynn's music therapy assessment tool (Glynn, 1992), Direct observation)	• Significant difference in verbal behaviour<br>• Residents used more verbal and non-verbal communication in the 20 minutes post-session<br>• Participants displayed predictable patterns of improvements in alertness, verbal or non-verbal interactions or energy. (statistics not provided)	Downs and Black 6 /21
**Raglio et al. (2008)**	Empathetic behaviour, Nonempathetic behaviour, smiling, body movement (Music therapy coding scheme (Raglio et al., 2006))	• Improved empathetic behaviours (f3,87=.10.37, p<0.0001; Cohen d= 0.61)<br>• Reduction in non- empathetic behaviours (f (3,87) =5.55, P=0.0015; Cohn d=1.8)<br>• Smiling (f (3,87) = 8.14, p<0.0001) and body movements (f (3,87) = 12.41, p<0.0001 increased<br>• Enhanced communicative relationship between resident and music therapist.	Downs and Black 20/25

Reviewers' final decision for question on Down and Blacks methodology quality checklist

Author	Q1	Q2	Q3	Q4	Q5	Q6	Q7	Q8	Q9	Q10	Q11	Q12	Q13	Q14	Q15	Q16	Q17	Q18	Q19
Engström et al. (2011a)	yes (1)	yes (1)	yes (1)	yes (1)	no (0)	yes (1)	yes (1)	No (0)	yes (1)	yes (1)	unable to determine (0)	unable to determine (0)	unable to determine (0)	n/a	no (0)	yes (1)	yes (1)	yes (1)	yes (1)
Lesta & Petocz (2006)	yes (1)	yes (1)	yes (1)	yes (1)	no (0)	yes (1)	yes (1)	no (0)	yes (1)	yes (1)	unable to determine (0)	unable to determine (0)	unable to determine (0)	n/a	no (0)	yes (1)	yes (1)	yes (1)	unable to determine (0)
Hammar et al. (2012)	yes (1)	yes (1)	yes (1)	no (0)	partically (1)	yes (1)	no (0)	No (0)	n/a	no (0)	unable to determine (0)	unable to determine (0)	unable to determine (0)	n/a	no (0)	yes (1)	n/a	unable to determine (0)	unable to determine (0)
Hsu el al. (2015)	yes (1)	yes (1)	yes (1)	yes (1)	partically (1)	yes (1)	yes (1)	yes (1)	yes (1)	yes (1)	unable to determine (0)	unable to determine (0)	unable to determine (0)	no (1)	no (0)	yes (1)	yes (1)	yes (1)	unable to determine (0)
Sambandham & Schirm (1995)	yes (1)	yes (1)	yes (1)	no (0)	no (0)	no (0)	no (0)	No (0)	unable to determine (0)	no (0)	unable to determine (0)	unable to determine (0)	unable to determine (0)	n/a	unable to determine (0)	no (0)	yes (1)	unable to determine (0)	yes (1)
Pollack & Namazi (1992)	yes (1)	yes (1)	yes (1)	yes (1)	no (0)	yes (1)	no (0)	No (0)	yes (1)	yes (1)	unable to determine (0)	unable to determine (0)	unable to determine (0)	n/a	no (0)	yes (1)	yes (1)	yes (1)	unable to determine (0)
Engstrom et al. (2011b)	yes (1)	yes (1)	yes (1)	yes (1)	n/a	yes (1)	n/a	No (0)	n/a	no (0)	unable to determine (0)	unable to determine (0)	unable to determine (0)	n/a	no (0)	yes (1)	n/a	yes (1)	yes (1)
Olderog Millard. & Smith (1989)	yes (1)	yes (1)	yes (1)	yes (1)	partically (1)	yes (1)	no (0)	No (0)	no (0)	yes (1)	unable to determine (0)	unable to determine (0)	unable to determine (0)	n/a	no (0)	yes (1)	yes (1)	yes (1)	unable to determine (0)
raglio et al. (2008)	yes (1)	yes (1)	yes (1)	yes (1)	yes (2)	yes (1)	yes (1)	No (0)	yes (1)	yes (1)	yes (1)	yes (1)	yes (1)	no (0)	yes (1)	yes (1)	yes (1)	unable to determine (0)	unable to determine (0)

Reviewer's Decision for CASP

author	Q1	Q2	Q3	Q4	Q5	Q6	Q7	Q8	Q9	Q10
Ridder & Gummesen (2015)	yes	yes	yes	can't tell	can't tell	no	yes	yes	yes	yes
Kydd (2001)	yes	yes	yes	can't tell	no	no	no	no	can't tell	Reasonable
Hsu et al. (2015)	yes	yes	can't tell	yes	yes	no	yes	yes	yes	Poor
Hammar et al. (2011a)	yes	yes	can't tell	can't tell	yes	no	yes	yes	yes	yes
Hammar et al. (2011b)	yes	yes	yes	no	yes	yes	can't tell	yes	yes	yes
Hammar et al (2010)	yes	yes	yes	can't tell	yes	yes	yes	yes	yes	yes
Gotell et al (2012)	yes	yes	yes	can't tell	yes	can't tell	yes	yes	yes	Reasonable
Gotell et al (2009)	no	yes	can't tell	can't tell	yes	no	yes	yes	yes	yes
Götell et al. (2002)	can't tell	yes	yes	can't tell	yes	yes	yes	yes	yes	Reasonable
Clare et al. (2019)	yes	yes	yes	no	yes	yes	yes	yes	yes	yes
Campbell et al. (2017)	yes	yes	yes	can't tell	yes	no	yes	yes	yes	yes
Ridder & Aldridge (2005)	can't tell	yes	can't tell	can't tell	yes	can't tell	no	can't tell	yes	Reasonable
Dassa & Amir (2014)	yes	yes	yes	no	no	yes	can't tell	no	yes	Reasonable
kuot et al (2021)	yes	yes	yes	can't tell	no	can't tell	yes	yes	yes	Reasonable
Swall et al (2020)	yes	yes	yes	can't tell	yes	yes	yes	yes	yes	Reasonable

y	Intervention	Control	Sample size (females)	Participant characteristic	Study
			ualitative study design		
	**Music in Mind Programme**<br><br>Sessions provided by musicians to develop musical improvisation. Access to instruments. 20-30 minutes.	N/A	11 interviewed<br><br>Number of session participants not provided	1 Music therapist<br><br>4 Manchester Camerata musicians<br><br>2 Manchester Camerata organisational team<br>3 Activity workers<br>1 Care home manager<br>Residents (not interviewed)<br><br>Resident characteristics not reported<br><br>Interviewees characteristics not reported	Explora<br>Qualita
	**Music for Life Group**<br><br>Active live music by musicians. Improvising singing and musical instruments playing. 1 hour.	1 Recorded music listening	8 Residents (4)	Mean age 90<br><br>CDR between 2.5 and 3.0	Qualita
	**Group music therapy**<br><br>Singing Israel songs from 1930-1950. Conversation between songs to evoke memories and feelings. 45 minutes.	N/A	6 Residents (2)	Mean age of 79<br><br>MMSE between 7-20	Qualita
	**MTC**<br>Condition 2- background music during personal morning care.<br><br>Condition 3 - Carers sang during personal care. 6 to 22 minutes.	Usual morning personal care	10 Residents (8)<br><br>5 Carers (5)	Residents- mean age 84 years old, MMSE score between 0-12, mean time at facilities 3 years 1 month.<br><br>Carers- licensed practical or mental health nurses, mean age 29 years old, fluent in Swedish, mean time in geriatric care 10 years	Qualita
	**MTC**<br><br>Condition 2- background music during personal morning care.<br><br>Condition 3- Carers sang during personal care. 6 to 22 minutes.	Usual morning personal care	9 Residents (7)<br><br>5 Care staff (5)	Residents-, MMSE mean 1, mean time at facilities 3 years 1 month<br><br>Carers- licensed practical nurses or mental health nurses, all but 1 born in Sweden, mean time in geriatric care 10 years	Qualita
	**MTC during person transfer**<br><br>Carers singing during transfer.	Usual transfer	19 Residents<br><br>17 Carers<br><br>9 Carers interviewed (8)	Resident- severe dementia, age & gender not reported<br><br>Carers - aged between 22- 55, worked in geriatric care between 1- 30 years	Qualita
	**MTC**<br><br>Carers singing during personal morning care	Usual morning personal care	10 Residents (6)<br><br>6 Carers (6)	Residents- age not reported, MMSE mean 3.3<br>Care- aged between 31-54, 4 nurse assistants, 2 nurse aids, working in dementia care between 2.5- 30 years.	Qualita

.	**MTC**<br><br>Carers singing during morning personal care	Usual morning personal care	10 Residents (6)<br><br>6 Carers (6)	Residents- age not reported, MMSE mean 3.3 Carers- aged between 31-54, 4 nurse assistants, 2 nurse aids, working in dementia care between 2.5- 30 years.	Qualita
.	**MTC**<br><br>Carers singing during personal morning care	Usual morning personal care	10 Residents (6)<br><br>6 Carers (6)	Residents-age not reported, MMSE mean of 3.3 Carers- aged between 31-54, 4 nurse assistants, 2 nurse aids, working in dementia care between 2.5-30 years.	Qualita
rk	**1:1 music therapy**<br><br>1:1 Singing, music playing and listening	N/A	1 Resident (0)	64 years old, moderate to severe dementia	Explora case stu
	**Group and 1:1 music therapy**<br><br>1:1 session - playing individuals chosen music, singing, piano playing.<br><br>30 minutes<br><br>Group sessions - weekly theme using music and singing to reminisces, exercise and socialise.	N/A	1 Resident (0)	88 years old<br><br>Dementia severity not reported	Case stu
	**1:1 music therapy**<br><br>Therapist singing and skills including holding, containing, validation and empathy.	N/A	1 Resident (1)	Early 70s, MMSE score 0, lived in the facility for 10 months	Case stu
a	**Personalised music playlist**<br><br>Families provided list of songs for the resident. Used an iPod device with headphones during the intervention. Listened to the playlist daily. 30 minutes.	N/A	10 Residents<br><br>15 Carers	Resident- mean 81 (67-93 years)<br><br>Gender not reported<br><br>Staff characteristics not reported	Qualita
.	**Staff training in Music in Caregiving**<br><br>Lecture provided on how to choose music for caregiving. How to make sessions person-centred and when to use music. After the lecture, staff used the techniques with a resident in their care home.	N/A	30 Carers (29)	Carers- aged 24-63<br><br>Resident characteristic not reported	Qualita
			**Mixed Methods**		
	**1:1 Music therapy**<br><br>Interactive music therapy skills including cueing, improvisation, joint music-making. Instruments available. Talking for reminiscence. Non-verbal communication used to express cues. 30 minutes	N/A	17 Residents (16)<br><br>10 Carers (7)	Residents- mean age 84, GDS mean 5.89 in music therapy, GDS mean 5.50 in control, mean time in facility 20 months.<br><br>Carers - mean age 38, mean of employed 32.33 months in music therapy and 23.33 months in control	Mixed r cluster random control

	**Quantitative study design**				
	**MTC**<br><br>Carers singing during personal morning care	Usual morning personal care	1 Resident (1)<br><br>1 Carers	Resident- 86 years old, MMSE score 4, lived at the facility for 5 years.<br><br>Carers not reported	A single<br>AB
a	**Group music therapy**<br><br>Music therapist provided songs to sing considering residents' sundowning. 30 minutes	N/A	4 Residents (4)	Aged between 80 to 97, MMSE less than 13, lived at the facility for minimum of 5 months	Before/
	**Group singing**<br><br>Researcher played a guitar while participants sang. 30 minutes	Discussion group researcher presented visual props to encourage group discussion. 30 minutes	10 Residents (7)	Mean age 81.4	Reversa<br>(ABAB/<br>repeate<br>measur
	**1:1 Music therapy**<br><br>Individuals preferred music activities- singing, moving, playing instruments. Reminiscence. 20 minutes	N/A	8 Residents (5)	Mean age 76.8<br>MMSE mean 7	Before/
	**MTC**<br><br>Carers humming during mealtimes .15 and 21 minutes.	Usual mealtime care	1 Resident (1)<br><br>1 Caregiver (1)	Resident-- 87 years old, MMSE score 0, time at facility 16 years<br><br>Carers- 51 years old, assisted nurse, worked in dementia care for 31 years, worked at facility for 6 years	ABA ca<br>study
	**MTC**<br><br>Carers singing during personal care	Usual morning personal care	10 Residents (6)<br><br>10 Carers	Residents- mean age 81.3, MMSE mean score 3.3, mean average time at facility 24.5 months<br><br>Staff characteristics not reported	Repeate<br>measur
	**Group music sessions**<br><br>60 minutes no other information given	N/A	19 Residents<br><br>Staff sample size not reported	Mean age 83, MMSE mean 7.1<br><br>Gender not report<br><br>Staff characteristics not reported	before/
	**Music therapy**<br><br>Non-verbal music therapy using rhythmical and melodic instruments to promote intersubject communication. 30 minutes	Educational and entertainment activities (personal care, lunch, bath, reading newspaper, playing cards)	59 residents (50)	Mean age 85.8 in control and 84.4 in experimental<br><br>MMSE - 10.7 in control and 11.1 intervention	Non-<br>random<br>control

Table presents author, year of publication, intervention, sample size and characteristic, study design and length of study
